# Investigating the role of morningness/eveningness in physical activity engagement

**DOI:** 10.1080/21642850.2022.2136183

**Published:** 2022-10-18

**Authors:** Lauren Nicholson, Barbara Mullan, Caitlin Liddelow

**Affiliations:** a School of Population Health, Curtin University, Perth, Australia; b Enable Institute, Curtin University, Perth, Australia; c WACPRU, Curtin University, Perth, Australia; d Global Alliance for Mental Health and Sport, School of Psychology, University of Wollongong, Wollongong, Australia

**Keywords:** Physical activity, morningness/eveningness, planning, motivation, habit

## Abstract

**Objective:**

Despite being aware of the positive health-related outcomes of physical activity, many people remain inactive. The aim of this study is to apply a combination of constructs from the health action process approach and self-determination theory, as well as habit and morningness/eveningness, to predict physical activity engagement.

**Methods:**

A prospective design was used to collect data from 136 participants (16–64 years old), at two-time points, one week apart. The sample consisted of 99 women, 36 men and 1 individual who identified as non-binary. Participants preferred time-of-day was measured using the Morningness-Eveningness Stability Scale (MESSi), while physical activity engagement was measured using the International Physical Activity Questionnaire (short-version). Two hierarchical, multiple regressions were conducted, to predict motivation to engage and to directly predict physical activity engagement. Furthermore, a mediation analysis was conducted to determine the effect of planning on physical activity engagement.

**Results:**

Results showed that younger individuals and those with greater self-efficacy were more motivated to engage while planning directly predicted physical activity engagement. However, morningness/eveningness did not significantly predict engagement. Additionally, planning was found to mediate the motivation-engagement relationship.

**Conclusion:**

This study demonstrates how planning influences individuals’ physical activity engagement, as well as the role self-efficacy and age play in their motivation to engage. Even though morningness/eveningness was not an important predictor, behaviour change techniques related to action planning and the use of multi-component approaches to behaviour change, could be used in interventions focused on increasing individuals’ physical activity engagement.

Physical activity is regarded as a complex behaviour consisting of many behavioural components and routines that need to be maintained long-term (Mullan & Novoradovskaya, 2018). Frequent physical activity is important for reducing poor health outcomes, such as cardiovascular disease and obesity (Hisler et al., 2017). Despite most people being aware of the positive health-related outcomes of physical activity, many people struggle to engage consistently (Wilson et al., 2012). For example, only 55% of Australian adults meet the recommended 150–300 min of moderate physical activity per day (Department of Health, 2021). Research has been conducted to understand the factors that prevent engagement, with a systematic review assessing the effectiveness of psychosocial factors in improving long-term engagement, finding that consistent physical activity engagement was associated with goal setting and feedback related to the behaviour (Tierney et al., 2012). However, studies such as these often exclude individual differences, such as preferred time-of-day (i.e. whether someone is more of a ‘morning person’ or ‘evening person’; Hisler et al., 2017).

Morningness/eveningness is an individual's preferred time-of-day, based on their peak functionality and internal body ‘clock’ (circadian rhythm; Adan et al., 2012). Therefore, this individual difference may impact individuals’ external schedules, for example, when they prefer to engage in physical activity (Montaruli et al., 2021). Understanding the role of morningness/eveningness in a physical activity context appears to be an evolving field. Previous research has shown that time-of-day plays an important role in physical activity behaviour and training habituation (Blazer et al., 2020). Research investigating the effects of preferred and non-preferred training times on motivation, found it was higher at individuals’ preferred time-of-day (Blazer et al., 2020). Additionally, a study looking at the effect of morningness/eveningness on physical activity exertion and performance, found that morning-oriented individuals exerted more effort in the afternoon session compared to the morning session, whereas the evening-type people showed the opposite (Mulè et al., 2020). However, no difference was found for both morning-types and evening-oriented individuals between morning and afternoon sessions (Mulè et al., 2020). Furthermore, Roveda et al. (2020) also found, in an adolescent population, that morning-types performed better physically in the morning sessions, whereas evening-orientated individuals performed better in the evening. Most of the previous literature investigating morningness/eveningness in a physical activity context has been conducted in a controlled environment (e.g. participants performing a single activity during a specific time block). In order to advance this, the current research took a more naturalistic approach by exploring the role morningness/eveningness plays in individuals’ normal physical activity routines. Furthermore, despite morningness/eveningness being shown to be important in behaviours such as physical activity, it is not often explored as a part of health psychology models. This lack of integration may suggest that when investigated congruently, morningness/eveningness could be more or less important than other established factors, such as planning. Two commonly applied theories to the study of physical activity are the health action process approach and self-determination theory.

The health action process approach provides a theoretical framework for improving and predicting health-related behaviours such as physical activity engagement (Maxwell-Smith et al., 2018). One component is self-efficacy, which refers to how confident a person is in their ability to perform a behaviour (Presseau et al., 2017). Previous literature suggests that individuals who have an intention and high self-efficacy are more likely to limit sedentary behaviour (Maher & Conroy, 2016). Previous literature has also explored how intention differs, based on an individual’s age. Alley et al. (2018) found that inactive older adults were less likely to have an intention to increase their physical activity engagement, compared to inactive younger adults.

Another component is planning, which involves knowing when, where and how to perform the desired behaviour (Teleki et al., 2021). Planning has been found to directly predict engagement in physical activity, suggesting that the greater extent to which individuals plan, the more likely they are to engage in their chosen physical activity (Teleki et al., 2021). This is because even though an individual might have an intention to perform a specific behaviour, they sometimes need goal-directed plans to actually execute that behaviour (Sheeran et al., 2005). Furthermore, planning has been found to be an important mediator, particularly in previous research that incorporates the health action process approach, as better planning helps individuals to translate their intentions into performing the behaviour (e.g. engaging in physical activity; Teleki et al., 2021).

Habit involves regular engagement in the same context for an association to develop between a cue and activity (Rhodes & Rebar, 2018). Habit has been shown to consist of two components: automaticity and routine (Ersche et al., 2017). Automaticity occurs as individuals learn to associate specific environmental cues with the initiation of behaviour, resulting in behaviours occurring without deliberation (Rhodes & Rebar, 2018). Additionally, routine is the regular execution of specific actions for a desired outcome (Wyckmans et al., 2020). Due to the complexity of physical activity, there have been many research debates as to whether it can truly be habitual (Rhodes & Rebar, 2018). However, Phillips and Gardner (2016) suggested that physical activity could be initiated habitually, even though performing these activities may require deliberate input.

Self-determination theory has previously often been used as a theoretical framework to explain the motivators of physical activity engagement (e.g. Mullan et al., 2021). Motivation has shown to successfully predict physical activity engagement in many studies (Maher & Conroy, 2016; Mullan et al., 2021; Teleki et al., 2021), therefore, this study integrated motivation with the components from the health action process approach, to see if it explains any additional variance, above and beyond the health action process approach variables.

## The current study

The aim of the current research is to apply constructs derived from the health action process approach, as well as the additional variables of motivation, habit and morningness/eveningness to predict engagement in physical activity.

Based on previous research, it is hypothesised:

(H1) Morningness/eveningness and self-efficacy will predict motivation to engage in physical activity.

(H2) Morningness/eveningness, motivation, planning and habit will predict engagement in physical activity.

(H3) According to Mulè et al. (2020), morning-oriented people show greater physical performance in the morning, compared to evening-type people. Therefore, we hypothesised that morningness/eveningness will moderate the relationship between motivation and engagement in physical activity, such that the association between motivation and engagement will be greater at high levels of morningness.

(H4) Based on previous research by Teleki et al. (2021), it is hypothesised that planning will mediate the relationship between motivation and physical activity engagement.

## Methods

### Participants

An *a* priori power analysis was conducted using G*Power (version 3.1.9.7) with a moderate effect size ($f^{2}\;$ =  .30 - .40; Keatley et al., 2012; Maher & Conroy, 2016) and eight predictors. As a result, this study required at least 59 participants. However, to account for anticipated attrition (of approximately 30%) and to obtain the required sample to detect a mediated effect (Fritz & MacKinnon, 2007), we aimed to recruit 150 participants. To be eligible, participants were required to understand written English, and be over the age of 16 years.

### Measures

In this study, physical activity was defined as any vigorous or moderate activity that was completed for recreation or exercise, for at least 30 min (Craig et al., 2003). Activities associated with commuting, housework, gardening, or team sports were excluded. Individuals often do not have the autonomy to decide when, where and how to engage (e.g. training sessions and fixtures are decided by coaches, clubs, and sporting associations). Therefore, to gain a better understanding of the role that planning, and habit may play, activities associated with team sport were excluded.

#### Morningness/Eveningness

Participants preferred time-of-day was measured using the Morningness-Eveningness Stability Scale (MESSi; Randler et al., 2016). This measure is divided into three subscales of five items: Morning Affect, Distinctness, and Eveningness. However, the current study only used the items related to Morning Affect (i.e. items 1–4 and 6) and Eveningness (i.e. items 5, 7 and 13–15). This decision was made as the focus of this study is not on the changes individuals’ experience in their psychological state throughout the day, but rather on their time-of-day orientation. Morning Affect measures alertness and energy levels after waking (e.g. ‘Assuming normal circumstances, how easy do you find getting up in the morning?’). Whereas, Eveningness measures affect and energy levels in the evening (e.g. ‘In general, how are your energy levels in the evening?’). All the items were measured on a five-point scale. Higher scores on Morning Affect indicates a preference for mornings (i.e. morningness), whereas higher scores on Eveningness shows an evening orientation (i.e. eveningness). The current study reported a Cronbach's alpha of .85 for the five-item Morning Affect subscale and .84 for the five-item Eveningness subscale.

#### Habit

The Creature of Habit Scale (COHS) was used, as it has been designed to measure the variations in the way individuals form habits, therefore, focusing on trait-based habitual tendencies instead of state-based ones (Ersche et al., 2017). This scale has been used in previous research exploring the association between a person’s tendency to form habits and health-related behaviours, such as alcohol consumption (Piquet-Pessôa et al., 2019). In order to advance this, the current study used the Creature of Habit Scale to measure people’s habitual tendencies in relation to being physically active. The COHS consists of 27-items, which are split into 16-items measuring ‘routine’ (e.g. ‘I find comfort in regularity’) and 11-items evaluating ‘automaticity’ (e.g. ‘I often find myself eating without being aware of it"). The Cronbach's alpha in the current study was .85. Both subscales were measured using a five-point scale ranging from 1 (definitely disagree) to 5 (definitely agree). Higher scores indicate stronger habitual tendencies.

#### Self-efficacy

The Spinal Cord Injury Exercise Self-Efficacy Scale (ESES) was used to measure self-efficacy in this context (Kroll et al., 2007). It was not necessary to adapt this scale, as the items were not worded specifically to assess individuals with a spinal cord injury. It includes 10-items evaluating individuals’ confidence in their ability to engage in physical activity (e.g. ‘I am confident that I can accomplish my physical activity goals that I set’). Items were answered using a four-point Likert-type scale, ranging from 1 (not at all true) to 4 (always true). The internal consistency of this measure in the current study was .84. Higher scores indicate greater self-efficacy (Newson & Kemps, 2007).

#### Motivation

Previous health action process approach literature has suggested that volition does not describe a person’s intention to engage, as well as motivation (Conner, 2008). Therefore, the current study combated this, by substituting the ‘volition’ components of the health action process approach with motivation. Motivation was measured by the Participation Motivation Questionnaire (PMQ; Gill et al., 1983). The PMQ includes 21-items asking participants to rate how often these reasons motivate them to engage in physical activity (e.g. ‘I enjoy physical activity’; Gill et al., 1983). Each of the 21-items was assessed using a five-point scale, from 1 (not at all) to 5 (always). The current study reported a great Cronbach's alpha of .81. Higher scores indicate higher levels of motivation (Gill et al., 1983).

#### Planning

Planning was assessed by the items used in Sniehotta et al's. (2005) research. Nine items assessed participants’ plans for where, when, and how they might engage in physical activity (e.g. ‘I have made a detailed plan regarding when to exercise’), and how they might cope with foreseen barriers (‘I have made a detailed plan regarding how to cope with possible setbacks"). Items were answered using a four-point scale ranging from 1 (completely disagree) to 4 (completely agree). Higher scores indicate better planning. The Cronbach's alpha in the current study was .91.

#### Physical activity engagement

Physical activity behaviour was evaluated at time one and time two using the shortened version of the International Physical Activity Questionnaire (IPAQ-S; Craig et al., 2003). In the original measure, six items explore a participant's physical activity over the past seven days (e.g. ‘During the last 7 days, on how many days did you do vigorous physical activities?’). However, in this study, we only used four items related to vigorous and moderate physical activity and removed two items related to walking as previous research has shown that walking is a type of moderate physical activity (Hoeger et al., 2008). The greater the amount of time reported engaging in physical activity, the greater the engagement in physical activity over the previous week.

### Procedure

The study was approved by the University’s Human Research Ethics Committee (HRE2017-0173). A prospective cross-sectional design was used to collect data between May to September 2021. Eligible participants were asked to complete an online questionnaire shared on the University’s participant pool and social media. An information sheet was provided, at both time points, and outlined the research and ethical considerations. Participants were required to provide consent, by checking a box, to proceed. Next, the participants were asked to provide an email address where they could be reached to complete the second phase. The first questionnaire took approximately 15 min to complete.

Seven days after the completion of time one, participants received an email containing a link to another online questionnaire. This questionnaire took approximately 5 min to complete.

### Data analysis

Descriptive statistics and two hierarchical multiple regressions were conducted in IBM SPSS Statistics 27. Additionally, bivariate correlations were conducted using the unstandardised predictors. Due to multicollinearity, the hierarchical multiple regressions and mediation analysis were conducted using standardised predictors. A missing values analysis was run at the individual item level. Only one case had at least one missing value identified, making the extent of missingness 0.70%. Little's MCAR test was non-significant, χ^2^ (93, *N* = 136) = 86.87, *p *= .659, indicating that this data was missing completely at random and therefore was imputed using expectation maximisation (Tabachnick & Fidell, 2013). Initially, we planned to control for all the demographic variables; however, upon inspection of the correlations, only age significantly correlated with motivation. Therefore, age was the only covariate in regression one. Furthermore, none of the demographic variables correlated with engagement in physical activity (time two); therefore, there were no covariates in regression two. For the first regression, the covariate age was entered at step 1, all the variables were added in steps two to four (morningness, eveningness, self-efficacy). For the second regression, all the variables were entered in steps one to five (morningness, eveningness, motivation, planning, habit). At step six, the interactions (motivation x planning, motivation x morningness, motivation x eveningness) were entered.

**Figure 1.**
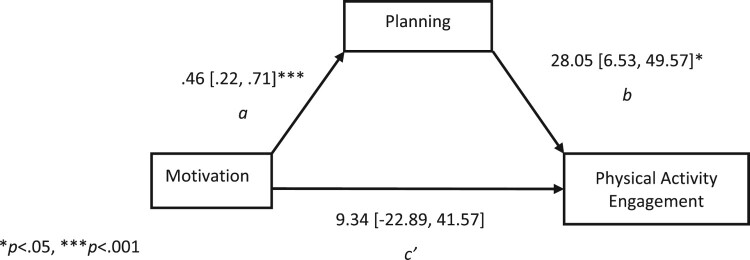
Full Mediation of the Relationship Between Motivation and Physical Activity Engagement via Planning. *p<.05, ***p<.001.

Furthermore, a mediation analysis was conducted using PROCESS (version 4.0) extension of IBM SPSS Statistics 27. Mediation analyses are concerned with investigating the effect of a predictor (i.e. motivation) in terms of how it accounts for the variance in the outcome variable (i.e. physical activity engagement; Allen et al., 2019). However, this is done by considering a mediating variable (i.e. planning). In the current study, a mediation analysis will help to determine whether the effect of motivation on physical activity engagement is indirect (i.e. the effect on the outcome variable through the mediator. Paths a and b) or direct (i.e. the effect on the outcome variable without the mediator. Path c’; Hayes, 2018) (see Figure 1). A mediation analysis can result in a partial mediation, where the mediator only accounts for some of the variance between the predictor and the outcome variable, or a full mediation model, where the mediator explains all the variance between the predictor and the outcome variable (Hayes, 2018).

## Results

### Participants

A total of 248 participants completed time one, and 140 participants completed time two (attrition of 43.55%). Responses were excluded if they did not complete time two, did not meet eligibility criteria, failed two attention check questions and if more than 30% of the survey was not completed. The final sample size of 136 participants provided adequate power, ranging from .996 to .999, *F*(_11, 124_) = 1.87. Participants’ age ranged from 16 to 64 years (*M* = 34.71, *SD* = 13.68), with 72.80% women (*n* = 99), 26.50% men (*n* = 36) and 0.70% identifying as non-binary (*n* = 1). Furthermore, 50% of participants reported they engaged in a mixture of cardio and weight/strength activity (*n *= 68), 39.70% engaged in only cardio-based physical activity (*n* = 54), 8.1% only engaged in weight/strength-based activity (*n* = 11), and 2.2% reported they engaged in neither (*n *= 3). The average amount of time spent per week engaging in physical activity was approximately 127 min at time one and 105 min at time two. Table 1 shows the means and standard deviations, whereas Table 2 contains the correlations between the variables used in the analyses.

**Table 1.** Descriptive Statistics of Physical Activity Engagement, Morningness, Eveningness, Habit, Self-efficacy, Motivation, Planning and Age.

	*M (SD)*
1. PA Engagement (T1)	127.71 (± 107.59)
2. Morningness	18.41 (± 3.90)
3. Eveningness	14.03 (± 4.23)
4. Habit	3.31 (± .49)
5. Self-Efficacy	3.31 (± .43)
6. Motivation	3.84 (± .43)
7. Planning	2.65 (± .64)
8. PA Engagement (T2)	105.68 (± 78.88)
9. Age	34.71 (± 13.68)

Note*:* PA = physical activity (in minutes); T1 = time one; T2 = time two.

**Table 2.** Correlations of Physical Activity Engagement, Morningness, Eveningness, Habit, Self-efficacy, Motivation, Planning and Age.

	2	3	4	5	6	7	8	9
1. PA Engagement (T1)	−.04	.10	.02	.16	.06	.08	.45***	.10
2. Morningness	−	−.52***	−.18*	.30***	.08	.08	.05	.35***
3. Eveningness		−	.01	−.17*	−.01	−.07	.02	−.14
4. Habit			−	−.31***	.09	.10	−.13	−.32***
5. Self-Efficacy				−	.29***	.29***	.28***	.17*
6. Motivation					−	.31***	.12	−.18*
7. Planning						−	.24***	−.05
8. PA Engagement (T2)							−	.11
9. Age								−

Note*:* PA = physical activity (in minutes); T1 = time one; T2 = time two; 2 = morningness; 3 = eveningness; 4 = habit; 5 = self-efficacy; 6 = motivation; 7 = planning; 8 = engagement in PA; 9 = age.

*p<.05, ***p<.001.

### Predicting motivation to engage in physical activity

In step one, age was controlled for and accounted for a significant 3.10% of variance in motivation. At step two, morningness accounted for an additional non-significant 2.20%. In step three, eveningness accounted for an additional, non-significant 0.30% of variance. Self-efficacy was added in step four and accounted for an additional significant 8.9%. In combination, the four predictors explained 14.4% of variance in motivation *R*^2^ = .14, *F*(_1, 131_) = 5.51, *p* < .001. However, age (*p* = .004) and self-efficacy (*p* < .001) were the only significant predictors. According to Cohen’s (1988) conventions, this is classified as a medium effect (f^2^ = .18) (see Table 3).

**Table 3.** Unstandardised (B) and Standardised (β) Coefficients, and Squared Semi-Partial Correlations (sr^2^) for Each Predictor Variable Entered into a Regression Predicting Motivation to Engage in Physical Activity.

	Variable	*B* [95% CI]	β	*sr*^2^	*p* value	*R*^2^	Δ*R*^2^	*F*	Δ*F* [*df*1, *df*2]
Step 1*					.042	.03	.03	4.22	4.22 [1, 134]
	Age	−.08 [−.15, −.00]*	−.18	.03	.042				
Step 2*					.028	.05	.02	3.67	3.06 [1, 133]
	Age	−.10 [−.17, −.02]*	−.23	.05	.012				
	Morningness	.07 [−.01, .14]	.16	.02	.083				
Step 3					.058	.06	.00	2.56	.36 [1, 132]
	Age	−.10 [−.18, −.02]*	−.21	.05	.011				
	Morningness	.08 [−.01, .17]	.19	.02	.072				
	Eveningness	.03 [−.06, .11]	.06	.00	.547				
Step 4***					<.001	.14	.09	5.51	13.65 [1, 131]
	Age	−.11 [−.18, −.04]*	−.26	.06	.004				
	Morningness	.05 [−.04, .13]	.12	.01	.293				
	Eveningness	.03 [−.05, .11]	.07	.00	.475				
	Self-efficacy	.13 [.06, .21]***	.31	.09	<.001				

Note*: B* = unstandardised coefficient; CI = confidence interval; β = beta (standardised coefficient); *sr*^2^ = squared semi-partial correlation coefficient.

*p<.05, ***p<.001.

### Predicting physical activity engagement

At step one, morningness explained a non-significant 0.30% of variance. In step two, eveningness accounted for an additional non-significant 0.30%. At step three, motivation accounted for an additional non-significant 1.3% of variance. Planning was added in step four and accounted for an additional non-significant 4.7% of variance. At step five, habit accounted for a non-significant 2.2%. Lastly, in step six, the interactions accounted for a significant 2.7% of variance. In combination, the eight predictors explained 11.5% of variance, *R*^2^ = .12, *F*(_3, 127_) = 2.06, *p* = .044. However, planning (*p* = .009) was the only significant predictor of physical activity engagement. According to Cohen’s (1988) conventions, this is classified as a small effect (f^2^ = .13) (see Table 4).

**Table 4.** Unstandardised (B) and Standardised (β) Coefficients and Squared Semi-Partial Correlations (sr^2^) for Each Predictor Variable Entered into a Regression Predicting Physical Activity Engagement.

	Variable	*B* [95% CI]	β	*sr*^2^	*p* value	*R*^2^	Δ*R*^2^	*F*	Δ*F* [*df*1, *df*2]
Step 1					.536	.00	.00	.39	.39 [1, 134]
	Morningness	4.22 [−9.24, 17.68]	.05	.00	.536				
Step 2					.674	.01	.00	.40	.41 [1, 133]
	Morningness	6.85 [−8.91, 22.60]	.09	.01	.392				
	Eveningness	5.08 [−10.67, 20.83]	.06	.00	.525				
Step 3					.468	.02	.01	.85	1.76 [1, 132]
	Morningness	5.91 [−9.86, 21.68]	.08	.00	.460				
	Eveningness	4.64 [−11.08, 20.36]	.06	.00	.561				
	Motivation	9.06 [−4.45, 22.56]	.12	.01	.187				
Step 4					.061	.07	.05	2.31	6.59 [1, 131]
	Morningness	5.31 [−10.15, 20.76]	.07	.00	.498				
	Eveningness	5.53 [−9.89, 20.95]	.07	.00	.479				
	Motivation	3.58 [−10.30, 17.47]	.05	.00	.611				
	Planning	18.01 [4.13, 31.90]*	.23	.05	.011				
Step 5*					.033	.09	.02	2.51	3.16 [1, 130]
	Morningness	2.27 [−13.43, 17.97]	.03	.00	.775				
	Eveningness	4.19 [−11.18, 19.55]	.05	.00	.591				
	Motivation	4.55 [−9.27, 18.37]	.06	.00	.516				
	Planning	19.05 [5.23, 32.87]*	.24	.05	.007				
	Habit	−12.10 [−25.58, 1.38]	−.15	.02	.078				
Step 6*					.044	.12	.03	2.06	1.29 [3, 127]
	Morningness	.32 [−15.48, 16.12]	.00	.00	.968				
	Eveningness	5.54 [−9.94, 21.02]	.07	.00	.480				
	Motivation	−.18 [−14.86, 14.50]	−.00	.00	.981				
	Planning	19.04 [4.86, 33.22]*	.24	.05	.009				
	Habit	−13.45 [−27.10, −.20]	−.17	.03	.053				
	Motivation×planning	−10.19 [−22.26, 1.87]	−.17	.02	.097				
	Motivation×morningness	−2.83 [−16.95, 11.30]	−.04	.00	.693				
	Motivation×eveningness	−.42 [−17.01, 16.26]	−.01	.00	.961				

Note*: B* = unstandardised coefficient; CI = confidence interval; β = beta (standardised coefficient); *sr*^2^ = squared semi-partial correlation coefficient.

*p<.05.

### Mediation analysis

To investigate whether the relationship between motivation and engagement in physical activity is indirect via planning, a mediation analysis was conducted. The mediation model explained a significant unique proportion of variance in engagement in physical activity *R*^2 ^= .09, *F*(_1, 134_) = 13.91, *p* < .01. According to Cohen (1988), this is a small effect (f^2^ = .10). The direct effect of motivation did not significantly predict unique variance in engagement, *c*’ = 9.34, 95% CI [22.89, 41.57], *p* = .567. The indirect effect of motivation via planning did significantly account for unique variance in engagement, *ab* = 12.88, BootLLCI/BootULCI [2.90, 27.25], *p* < .05. The results indicate full mediation (Figure 1) of the relationship between motivation and physical activity engagement by planning (see Table 5). This suggests that individuals with better planning skills are more likely to translate their motivation to engage into performing their chosen physical activity.

**Table 5.** Unstandardised (B) Regression Coefficients, 95% Confidence Intervals (CI), and R-Squared Coefficients for Motivation, Planning and Physical Activity Engagement.

Variable	*B* [LLCI, ULCI]	*SE*
DV = Plan (*R*^2^ = .09)***		
Constant	.89 [−.48, 1.83]	.48
Motivation	.46 [.22, .70]	.12
DV = PA Engagement (*R*^2^ = .06)*		
Constant	−4.58 [−124.50, 115.35]	60.63
Motivation	9.34 [−22.89, 41.57]	16.29
Planning	28. 05 [6.53, 49.57]	10.88

Note*:* PA* *= physical activity; *B* = unstandardised regression coefficients; LLCI = bootstrapped lower level confidence interval; ULCI = bootstrapped upper level confidence interval; *SE* = standard error estimates.

*p<.05, ***p<.001.

## Discussion

The current study explored constructs derived from the health action process approach in combination with motivation, habit and morningness/eveningness, to investigate the predictors of physical activity engagement. The results showed self-efficacy and age were the only significant predictors of motivation to engage, whereas planning was the only significant predictor of physical activity engagement. Furthermore, planning was found to mediate the relationship between motivation and engagement in physical activity.

### Predicting motivation to engage in physical activity

Our hypothesis that morningness/eveningness and self-efficacy would predict motivation to engage in physical activity when controlling for age, was partially supported as results showed that self-efficacy and age were the only significant predictors. This suggests that greater self-efficacy leads to increased motivation to engage in physical activity, in line with previous research (Tierney et al., 2012). Therefore, health professionals and personal trainers could look to increase self-efficacy by encouraging physical activity engagement with friends/family, as they provide support by conveying knowledge, and facilitating safe activity (Steltenpohl et al., 2019). The current findings also provide further theoretical support for the importance of self-efficacy in understanding health behaviour.

The results showed younger adults were more motivated to engage in physical activity than older adults, suggesting that age is an important predictor of motivation. This is in line with previous literature showing inactive older adults to be less motivated to increase their physical activity than inactive younger adults (Alley et al., 2018). Literature in this area shows that social interaction can act as a motivator for older adults to engage (Kritz et al., 2021). Health professionals could therefore encourage older adults to engage in activities that promote social connection to improve their adherence to physical activity (Steltenpohl et al., 2019). Additionally, older adults may be less motivated to engage in physical activity as they may have physical injuries or degeneration that prevent them from being active (e.g. arthritis, poor mobility, weakened muscles). With this in mind, older people may also need the assistance of a walking frame or a wheelchair in order to move around. This is likely to decrease their motivation to be physically active as it limits the environments they can access and may mean they have to rely on others to help them move around. Therefore, health professionals, family members, or nurses could help older individuals set out times for when they can be physically active, so that they are able to help, as well as think about environments that are easily accessible (e.g. smooth paving in the neighbourhood, or an exercise pool; Costello et al., 2011).

### Predicting physical activity engagement

Our hypothesis that morningness/eveningness, habit, planning and motivation would predict physical activity engagement was partially supported. Results showed that planning was the only significant predictor of physical activity. This aligns with previous literature using the health action process approach, as it shows planning to be a strong predictor of individuals’ engagement and maintenance of physical activity, despite potential barriers (Teleki et al., 2021). This provides further theoretical support for the importance of planning in behaviour change. Health professionals or personal trainers could implement the ‘action planning’ behaviour change technique to increase physical activity engagement for clients who are inactive (Michie et al., 2013). This behaviour change technique can be implemented by developing actionable plans (i.e. when, where and how) that encourage engagement (Michie et al., 2013).

Additionally, the results showed that habit was not a significant predictor of physical activity engagement. Previous literature debates the true habitual nature of physical activity (Rhodes & Rebar, 2018), and our finding was not in line with more recent habit research which suggests that physical activity engagement can be initiated habitually (Phillips & Gardner, 2016). A potential explanation could be that the study was slightly underpowered. According to Cohen (1988), there was a small effect ($f^{2}$ = .13) between habit and physical activity engagement. Future research could explore the role of a person’s tendency to habitually engage in physical activity by investigating whether routine is more important than automaticity. However, to investigate the role habit plays specifically in physical activity engagement, future research could also continue to use the more common Self-Report Habit Index (Verplanken & Orbell, 2003), as it measures habit strength by focusing on state-based automaticity (i.e. for a specific behaviour) rather trait-based.

Furthermore, results showed that motivation was not a significant predictor of physical activity engagement. This is not in line with the literature, as it has often been used to predict physical activity engagement (Deci & Ryan, 2000). Based on this, motivational approaches to behaviour change might not be suited to each individual or behaviour. The implementation of a multi-component approach to behaviour change might be more beneficial, as it would allow for an individualised action plan to be developed based on the person's goals, beliefs, and expectancies, rather than just on motivational factors (Lachman et al., 2018). An additional explanation could be that motivation is not the most important predictor of behavioural engagement. This is not uncommon, with previous research also finding other factors such as attitudes (Ogden et al., 2007) and cues to action (Liddelow et al., 2021) were more important predictors of health behaviour engagement, compared to motivation. Previous research has shown that enjoyment plays an important role in individuals consistently engaging in physical activity (Carraro et al., 2014; Jekauc, 2015). Therefore, future research could explore enjoyment as part of health models, to see if it is a more proximal predictor of physical activity behaviour.

### Role of morningness/eveningness

In contrast to our hypothesis, results showed that morningness/eveningness did not predict motivation to engage or engagement in physical activity. This suggests that engaging in physical activity during an individual's preferred time-of-day may not be important in this sample. The current results appear to contrast those of Blazer et al. (2020), who found individuals had higher motivation to engage during their preferred time-of-day. Furthermore, Mulè et al. (2020) suggested that individuals performed physical activities better at their preferred time-of-day. Based on other previous research (Díaz-Morales & Randler, 2017), an explanation for our finding could be that during an individual’s preferred time-of-day, they might engage in activities that require peak cognitive functioning (e.g. studying), rather than peak physical functioning. Future research should explore this by investigating whether cognitive load predicts if individuals engage in more physical or cognitive functioning activities during their preferred time-of-day. A further explanation might be that external events (e.g. taking care of children, shift work, seasonal changes) could inhibit people from engaging in physical activity (Sechrist et al., 1987). Future research could investigate whether this barrier prevents people from engaging in physical activity at their preferred time-of-day. This could be done by incorporating an open-ended question or conducting a qualitative study, exploring why individuals think they do not engage in physical activity at their preferred time-of-day.

Furthermore, our hypothesis that morningness/eveningness would moderate the relationship between motivation and physical activity engagement, was not supported. It appears that no other previous research has explored this moderating effect. Additionally, the present study also found that planning does not significantly moderate the relationship between motivation and physical activity engagement. This is not in line with previous research showing the motivation-activity relationship was stronger when individuals plan to a greater extent (de Bruijn et al., 2012). These two results may suggest other variables moderate the motivation-engagement relationship. Based on this, future research could explore the effect other predictors, such as self-control, have on the motivation-engagement relationship.

### Planning mediation

Our results showed that planning significantly mediated the relationship between motivation and physical activity engagement, supporting previous literature that utilises the health action process approach (Scholz et al., 2008). This mediation suggests motivation to engage predicts the extent to which an individual plans their engagement, and subsequently, this predicts their engagement in physical activity. For instance, a highly motivated individual will plan to a greater extent and, therefore, engage in physical activity more consistently. Health professionals could help individuals improve the extent to which they plan, by implementing goal-setting theory into the planning of their engagement in physical activity (Swann et al., 2022). This can be done by assessing the individual’s commitment, knowledge, facilities and ability to perform their desired physical activity (Swann et al., 2022). Based on this initial assessment specific plans can be created to achieve their physical activity goals (Swann et al., 2022).

### Strengths and limitations

Previous research investigating the role of morningness/eveningness in the context of physical activity has occurred in controlled environments, requiring participants arrive at specific times during the morning, afternoon and evening, to perform a specific exercise (e.g. bench press, fitness tests; Blazer et al., 2020; Mulè et al., 2020). In contrast, the current study has a more naturalistic design. This is a strength, as it provides a truer representation of how important morningness/eveningness is in individuals engaging in physical activity. Overall, there is not a lot of research that explores this individual difference in the context of physical activity. Therefore, another strength of the current study is that it contributes to the growing body of research in this field. This is important as it can help health professionals and individuals see what could be inhibiting them from engaging in physical activity on a regular basis.

This study is not without its limitations. The current study measured physical activity subjectively with the shortened version of the International Physical Activity Questionnaire, which is a well-validated measure in physical activity research. However, we were unable to measure physical activity objectively. Only having self-report data could suggest the amount of physical activity that each participant reported is not an accurate reflection of their physical activity engagement. In order to reduce this potential inaccuracy, future research could look at collecting physical activity data via participants’ own fitness watches. Participants sending screenshots of their physical activity from these devices could act as supporting evidence for any subjective data that might be collected. This could allow researchers to gather data that may be a better representation of an individual’s engagement.

## Conclusion

The aim of this study was to apply constructs from the health action process approach and self-determination theory, with the additions of habit and morningness/eveningness, to predict physical activity engagement. The current study showed that self-efficacy and age are important predictors of motivation to engage in physical activity. Furthermore, physical activity engagement can be predicted by planning. Even though morningness/eveningness was not a significant predictor, these findings could guide behaviour change techniques focused on implementing actionable plans, the formation of physical activity routines and promoting social interaction. Furthermore, it could guide interventions aimed at facilitating an increase in physical activity engagement.

## Data Availability

The data that support the findings of this study are openly available in OpenScience Framework at http://doi.org/10.17605/OSF.IO/ZVY9P.
